# Maturation of subtilisin-like protease NbSLP1 from microsporidia *Nosema bombycis*

**DOI:** 10.3389/fcimb.2022.897509

**Published:** 2022-08-15

**Authors:** Rong Wang, Qingyan Li, Fangyan Liu, Xiaoqun Dang, Quan Sun, Xiaotian Sheng, Mingyu Hu, Jialing Bao, Jie Chen, Guoqing Pan, Zeyang Zhou

**Affiliations:** ^1^ State Key Laboratory of Silkworm Genome Biology, Chongqing Key Laboratory of Microsporidia Infection and Control, Southwest University, Chongqing, China; ^2^ College of Life Sciences, Chongqing Normal University, Chongqing, China

**Keywords:** microsporidia, *Nosema bombicys*, subtilisin-like protease, autoproteolysis, germination

## Abstract

Microsporidia are obligate intracellular parasites and possess a unique way of invading hosts, namely germination. Microsporidia are able to infect almost all animal cells by germination. During the process, the polar tube extrudes from the spores within, thus injecting infectious sporoplasm into the host cells. Previous studies indicated that subtilisin-like protease 1 (NbSLP1) of microsporidia *Nosema bombycis* were located at the polar cap of germinated spores where the polar tube extrusion. We hypothesized that NbSLP1 is an essential player in the germination process. Normally, SLP need to be activated by autoproteolysis under conditions. In this study, we found that the signal peptide of NbSLP1 affected the activation of protease, two self-cleavage sites were involved in NbSLP1 maturation between Ala^104^Asp^105^ and Ala^124^Asp^125^ respectively. Mutants at catalytic triad of NbSLP1 confirmed the decreasing of autoproteolysis. This study demonstrates that intramolecular proteolysis is required for NbSLP1 maturation. The protease undergoes a series of sequential N-terminal cleavage events to generate the mature enzyme. Like other subtilisin-like enzymes, catalytic triad of NbSLP1 are significant for the self-activation of NbSLP1. In conclusion, clarifying the maturation of NbSLP1 will be valuable for understanding the polar tube ejection mechanism of germination.

## Introduction

Microsporidia, a group of obligate intracellular and unicellular fungi alike (Keeling and Fast, 2002), can infect almost all kinds of invertebrates and vertebrates, including immuno-competent and immuno-deficient people (Didier and Weiss, 2011) as well as economic animals, like silkworm, shrimp and honey bees (Mai et al., 2020). The life cycle of the parasite divides into three parts, namely the infective phase(environmental phase), proliferative phase, and sporogonic phase (Franzen, 2004). In the infective phase, the only extracellular stage, mature spores can be activated by some stimulus, then eject the polar tube and transport infective sporoplasm into host cells, i.e., germination (Han and Weiss, 2017). Germination of microsporidia is a significant infectious process for the parasites, although the mechanism has not been unrevealed up to now.

Previous studies have shown that subtilisin-like protease (SLP) widely exist in microsporidia. In *Paranosema locustae*, the microsporidia infecting migratory locust, SLP play roles in physiology of the spores instead of host-parasites interaction (Timofeev et al., 2014). SLP of *Encephalitozoon cuniculi* may be involved in spore development regulation (Ronnebaumer et al., 2006). Three subtilisin homologs, namely NbSLP1, NbSLP2-1, and NbSLP2-2, were found in *Nosema bombycis*, the first identified microsporium which infects *Bombyx mori*. NbSLP2 localized around spore walls, and could interact with cytoskeletal protein Nbseptin2 (Liu et al., 2017), while NbSLP1 showed apical localization features, and was activated after germination (Dang et al., 2013), which may imply the function in germination.

As a biological regulator, protease is involved in all sorts of biological processes in parasites. For SLP, these proteases always act as virulence factors in pathogenic microorganisms, involving in the formation of infective organelle, egress, and invasion in the host cells. In parasites, *Toxoplasma* and *Cryptosporidium* share the similar subtilisin SUB1, which acts as a pioneer for the parasite’s infection (Wanyiri et al., 2009; Saouros et al., 2012), while *Plasmodium falciparum* SUB1 processes membrane-anchored protein MSP1, which interacts with Red Blood Cell spectrin cytoskeleton, playing a role in the egress of merozoite from erythrocytes (Das et al., 2015). Besides, SUB2 and SUB3 play different roles in *Plasmodium* when infecting host (Barale et al., 1999; Withers-Martinez et al., 2004; Alam et al., 2013). In fungi, Kex2 of *Blastomyces albicans* is involved in pathogenesis when infecting host cells; SPM1 (subtilisin in *Magnaporthe oryzae*) is involved in multifunction infection, including infection-related germination, invasion, and growth (Oh et al., 2008; Saitoh et al., 2009). Subtilisin in bacteria is a significant virulence effector, which was demonstrated by knock-out of CylA in *Enterococcus faecalis* and IvaP in *Vibrio cholerae* (Howell et al., 2019; Tang et al., 2019).

Subtilisin is produced as a large precursor, containing a pro-peptide domain and catalytic domain, in which the pro-peptide domain served as an intramolecular chaperone to ensure the protease fold correctly, and as a temporary inhibitor to inhibit the protease activity (Takagi et al., 2001; Yabuta et al., 2001). In some biological processes, subtilisin is triggered by certain conditions, then autoprocesses to release the pro-peptide domain and become a mature enzyme.

The present research explores self-activation of heterologous-expressed NbSLP1 and identifies the two autoproteolytic sites and three active sites. It is hoped that this research will contribute to a deeper understanding of NbSLP1 maturation and help to explore the polar tube extrusion.

## Materials and methods

### Plasmid construction

Full length of *Nbslp1*(*FL-Nbslp1*) and a signal peptide deleted fragment of *Nbslp1* (*Pro-Nbslp1*) were amplified by PCR with the genome of *Nosema bombycis* preserved in our lab. Primers designed based on *Nbslp1* sequence from *N. bombycis* database (https://silkpathdb.swu.edu.cn/) are listed in **Table 1**. Recombinant plasmid pET30a-*FL-Nbslp1* and pET30a-*Pro-Nbslp1* were constructed by inserting *Bam*HI and *Sal*I restriction endonuclease digested pET30a vector.

**Table 1:** Primers for *Nbslp1* and mutants.

Primer ID	Primer sequence (5’-3’)	Length	Fragment
FL-Nbslp1-F FL-Nbslp1-R	CGggatccATGAATCTTGAATCAATTCCAAAT GCgtcgacATTTAATTGTTCATTTTCAA	1476 bp	*FL-Nbslp1*
Pro-Nbslp1-F Pro-Nbslp1-R	CGggatccGATAATTATATTGTGATGTTTAAA GCgtcgacATTTAATTGTTCATTTTCAA	1395 bp	*Pro-Nbslp1*
Nbslp1-MRIA^104^-F Nbslp1-MRIA^104^-R	AAGACAAGATGgctgctgctgctGATTTTAATTTAA TTAAATTAAAATCagcagcagcagcCATCTTGTCTT	1188 bp 245 bp	*Nbslp1-mutA-1*
Nbslp1-DFNL^108^-F Nbslp1-DFNL^108^-R	gctgctgctgctgctgctGCAAGTGATTATC GATAATCACTTGCagcagcagcagcagcagc	1182 bp 253 bp	*Nbslp1-mutA-2*
Nbslp1-MADFNL-F Nbslp1-MADFNL-F	GAATTTATCgcggcagccgctgctgctTTGTC GACAAagcagcagcggctgccgcGATAAATTC	1116 bp 308 bp	*Nbslp1-mutB*
Nbslp1-D^192^A-F Nbslp1-D^192^A-R	AGGTTTACGTTATCgccACAG TCGATACCTGTggcGATAACG	917 bp 497 bp	*Nbslp1-D^192^A*
Nbslp1-H^224^A-F Nbslp1-H^224^A-R	ATGAAAACGGAgccGGAACT AGTTCCggcTCCGTTTTCAT	818 bp 600 bp	*Nbslp1-H^224^A*
Nbslp1-S^413^A-F Nbslp1-S^413^A-R	AGGAACAgccATGGCAACTC GAGTTGCCATggcTGTTCCT	247 bp 1171 bp	*Nbslp1-S^413^A*

*a. restriction endonucleases BamHI (ggatcc) and SalI (gtcgac) were showed in lowercase letters; b. mutant sites were showed in underlined lowercase letters.

### Heterologous expression

Recombinant plasmids pET30a-*FL-Nbslp1* or pET30a-*Pro-Nbslp1* were transformed into *E. coli* BL21(DE3). The positive clones were inoculated in 5 mL LB medium containing 50 μg/mL of kanamycin at 37°C for more than 8 h. 300 μL of the cell suspension were transferred into 30 mL culture medium, shaken with 180 rpm at 37°C for 2-3 h, until reaching A_600_~0.6. Expression of His_6_-FL-Nbslp1-His_6_ or His_6_-Pro-Nbslp1-His_6_ was induced by adding 0.2 mM isopropyl β-D-thiogalactopyranoside (IPTG) to the culture medium, prior to inoculating at 16°C, 130 rpm for 20-24 h. 5 mL of culture were lysed by ultrasonication. Soluble and inclusion body fractions were separated by centrifugation at 13000 rpm for 10 min. Protein samples were subjected onto the SDS-PAGE and analyzed by Western blot.

### Protein Purification

The cultured medium of recombinant bacteria was collected (300 mL of culture medium). The supernatant was then ultrasonically fractured in 20 mL Binding buffer (2 mM KH_2_PO_4_, 10 mM Na_2_HPO_4_, 140 mM NaCl, 3 mM KCl, 20 mM imidazole, pH7.4) and centrifuged at 12000 rpm, 4°C for 10 min to isolate the supernatant, which was subsequently syringe-filtered using 0.22 μm filter. The filtrate was subjected to Ni-NTA Agarose (QIAGEN), washed with 20 mM and 50 mM imidazole, and purified protein was eluted by 500 mM imidazole. The recombinant protein then underwent the de-salting process, and was then dialyzed into PBS buffer overnight at 4 °C with constant stirring. The desalted protein was concentrated by freeze-drying and further purified by Gel filtration chromatography (HiLoad™ 16/600 Superdex™ 200 pg, GE Healthcare).

### Western blot

Protein samples were loaded on SDS-PAGE gels and transferred to PVDF membranes with Tris-Gly Transfer Buffer. The membranes were blocked with 5% skim milk in TBST buffer at room temperature for 1 hour, and then incubated overnight at 4°C with primary antibody and anti-His antibody (Roche, 1:1000 dilution in blocking solution). After 3 washes with TBST buffer, the membranes were incubated for 1 hour with Peroxidase Conjugated Goat anti-Mouse IgG, (H+L) (Sigma-Aldrich, 1:5000 dilution). The blots were detected with ECL western blot detection kit (Thermo Fisher).

### His-tag pull-down assay

To enable us to identify the protease and digestion site after self-shearing, His pull-down was performed following the instructions (ThermoFisher Scientific) to purify the recombinant proteins. After washing with TBS, *E. coli* pellets that express the recombinant proteins were suspended in 1 mL of ice-cold TBS and 1 mL of ProFound™ Lysis Buffer and incubated on ice for 30 min, then centrifuged to clarify crude lysate. 50 μL immobilized cobalt chelate into Handee™ Spin Column was washed 5 times with wash solution (1:1 wash solution of TBS : ProFound™ Lysis Buffer to which 4 M Imidazole Stock Solution was added to a final concentration of 40 mM imidazole). Polyhistidine-tagged fusion protein lysates were incubated with settled cobalt chelate resin at 4°C for 2 h with a gentle rocking motion on a rotary mixer. After 5 washes, 250 μL of 290 mM Imidazole Elution Buffer (63 μL of 4 M Imidazole Stock Solutionadded to 937 μL of wash solution per 1 mL Elution Buffer) was added into each spin column, then incubated at 4°C for 5 min, and finally elution was collected by centrifugation at 1250 rpm for 30 sec.

### Mass spectrometry analysis

Protein bands captured from Pull-down assay were collected in 1.5 mL centrifuge tubes. After reduction and alkylation treatment, Trypsin (mass ratio: 1:50) was added to the test samples for enzymatic hydrolysis at 37 conditions for 20 h. The enzymatic hydrolysis samples were desalted and lyophilized, then dissolved in 0.1%FA solution and stored at -20°C for later use. Mass spectrometry analysis: Solution A was an aqueous solution of 0.1% formic acid, and Solution B was an acetonitrile aqueous solution of 0.1% formic acid (84% acetonitrile). After the chromatographic column was balanced with 95% of Solution A, the sample was loaded from the automatic sampler to TRAP column. Mass spectral data collection: the mass charge ratio of polypeptides and polypeptide fragments was collected by the following methods: 20 fragment maps were collected after each full scan (MS2 scan); Mascot 2.2 software was used to retrieve the corresponding database for the original file of mass spectrometry test; and finally the identified protein result was obtained. The search database parameters are listed in **Table 2**.

**Table 2:** The retrieval parameter table of database.

Paraments	condition
Enzyme	Trypsin
Database	uniport_*Nosema_bombycis*_4624_20191014.fasta
Fixed modification	Carbamidomethyl (C)
Variable modification	Oxidation (M)
Missed Cleavage	2
Peptide Mass Tolerance	20 ppm
Fragment Mass Tolerance	0.1 Da
Filter by socore	>=20

### N-terminal sequence analysis

After being pre-run for 1 hour, protein captured from Pull-down assay was subjected onto an SDS-PAGE gel and then transferred to PVDF membrane (Roche) using CAPS Buffer (10 mM CAPS, pH 11). Target protein band was visualized with Coomassie Bright Blue R-250, and then cut off from the membrane and tested by the PPSQ Protein Sequencer (SHIMADZU). Raw data and chromatogram were analyzed by PPSQ DataProcessing/Labsolutions.

### Enzymatic activity

To assess the enzyme activity of purified recombinant protein, In-gel zymography was conducted (Lantz and Ciborowski, 1994; Seawell and Bose, 2021). Subtilisin purchased from Sigma (Sigma) was used as positive control. Protease samples in nonreducing sample buffer (2.5% SDS) was prepared without boiling. Electrophoresis of the samples on 12% SDS-polyacrylamide gel containing casein (1 mg/mL) was carried out on ice. After 2 washes with 2.5% TritonX-100 diluted in distilled water and 2 washes with 2.5% TritonX-100 diluted in TB buffer (50 mM Tris-HCl buffer, pH 7.4) for 10 minutes respectively, the gel was washed with TB buffer to remove SDS and TritonX-100. After incubation for 24-48 hours at 37 °C in TB buffer containing 10 mM CaCl_2_, the gel was fixed with fixing buffer (50% methanol and 50% acetic acid) for 10 minutes and stained by Coomassie blue R-250 for 30 minutes. Regions with enzyme activity appear as a clear colorless region against the blue background.

### Mutants

To conform the two autocleavage sites which identified by N-terminal sequence analysis, NbSLP1 mutants were designed. Before the experiments in the lab, homology modelling of NbSLP1 and its mutants was conducted to confirm the structures of the proteins with little variation. The target amino acid sequences were submitted in Swiss-Model (https://swissmodel.expasy.org/). With Pro-subtilisin E (PDB: 3whi.1) being the template of NbSLP1 and its mutants, 3D protein models were automatically generated and used to analyze the variation in position. After that, *Nbslp1* mutants (*Nbslp1^mut^*) were generated by PCR site-directed mutagenesis. The strategy of mutation is shown in **Figure 4A** and primers are listed in **Table 1**. Briefly, *Nbslp1^mut^* were generated by overlap extension PCR with mutant fragments as templates. Then recombinant plasmids pET30a-*Nbslp1^mut^* were constructed by the same methods as above.

## Results

### Identification of NbSLP1 autoproteolysis and cleavage sites

**Figure 1:**
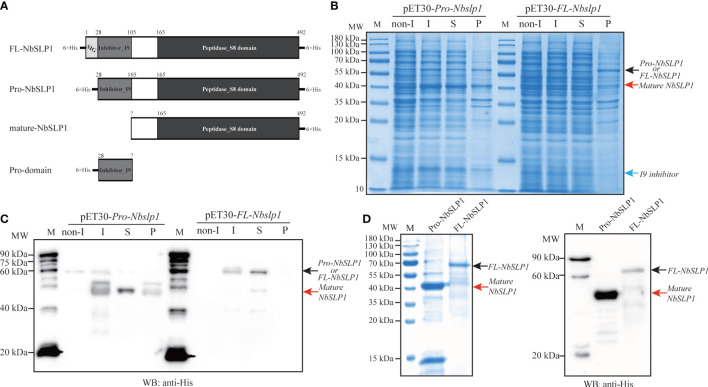
Heterologous expression analysis of Pro-NbSLP1 and FL-NbSLP1. **(A)** Predicted models of full-length NbSLP1 (FL-NbSLP1), signal peptide deleted NbSLP1 (Pro-NbSLP1), mature-NbSLP1 and Pro-domain (Inhibitor_I9 domain), His-tag is showed in thick lines; **(B)** SDS-PAGE analysis of recombinant expressed Pro-NbSLP1 and FL-NbSLP1 in *E*. *coli* BL21 (DE3). Lane M: premixed protein marker (10-180 kDa); **(C)** Western blot analysis of Pro-NbSLP1 and full-length NbSLP1. **(D)**: SDS-PAGE and Western blot analysis of purified protein extracted from *E*. *coli* BL21 (DE3) transformed with pET30-*pro*-*Nbslp1* and pET30-*FL-Nbslp1.* M: ECL marker (20-90 kDa); Full-length NbSLP1 and Pro-NbSLP1 are indicated by black arrows; mature NbSLP1 are indicated by red arrows and Pro-peptide (Inhibitor_I9 domain) is indicated by blue arrow. Primary antibody: anti-His antibody (1:1000 dilution); non I: none induced; I: IPTG induced; S: supernatant after ultrasonication; P: precipitate after ultrasonication.

**Figure 2:**
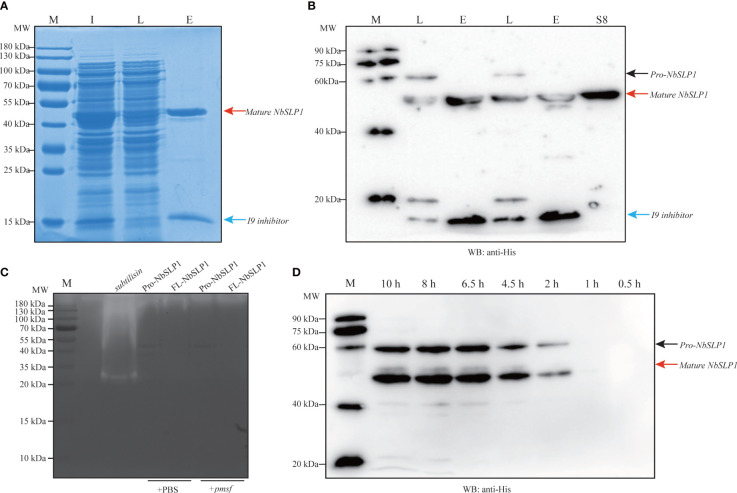
Identification of Pro-NbSLP1 autoproteolysis in *E. coli.* **(A)** SDS-PAGE analysis of Pro-NbSLP1 by pull-down assay. **(B)** Western blot analysis of Pro-NbSLP1 by pull-down assay. Lane M in **(A)** and **(C)**: premixed protein marker; Lane M in **(B)** and **(D)**: ECL marker; Lane I: crude lysate extracted from induced *E*. *coli* BL21 (DE3) transformed with pET30-*Pro*-*Nbslp1*; Lane L: Flow-through after combination with cobalt chelate resin; Lane E: Elution of Pro-NbSLP1 by Pull-down assay; Lane S8: NbSLP1-Peptidase_S8 peptide; Pro-NbSLP1 are indicated by black arrow; mature NbSLP1 are indicated by red arrows and Pro-peptide/I9 inhibitor are indicated by blue arrows. **(C)** Identification of enzymatic activity by casein zymography. Subtilisin was purchased from Sigma as positive control. Pro-NbSLP1 and FL-NbSLP1 adding PMSF (phenylmethylsulfonyl fluoride) were as inhibitor control. **(D)** Pro-NbSLP1 autoproteolysis at different hours post induction by IPTG.

In order to identify the autoproteolysis of NbSLP1, pET30-*Pro-Nbslp1* and pET30-*FL-Nbslp1* expression vector with a N-terminal His_6_-tag and a C-terminal His_6_-tag were constructed and transformed into *E. coli* BL21 (DE3) respectively (**Figure 1A**). Western Blotting analysis showed that His_6_-FL-NbSLP1-His_6_ was expressed and purified in soluble form with a molecular weight of ~61.83 kDa, indicated by a black arrow and light band at ~44.55 kDa, indicated by a red arrow, while His_6_-Pro-NbSLP1-His_6_ was expressed and purified in soluble form with a molecular weight of ~44.55 kDa, indicated by a red arrow, while inclusion bodies formed with two bands with a molecular weight of ~58.77 kDa and ~44.55 kDa (**Figures 1B**–**D**), which share a similar size with predicted Pro-NbSLP1 and mature-NbSLP1, respectively. To further affirm autoproteolysis of NbSLP1 in the prokaryotic expression system, pull-down assay was carried out to obtain putative His_6_-inhibitor_I9 and mature-NbSLP1-His_6_ (**Figures 2A**, **B**), then they were identified by mass spectrum. The results showed that the two bandsat ~44.55 kDa were both identified as Subtilisin-like serine protease, NbSLP1, which means that NbSLP1 could autoproteolyse twice in *E. coli* BL21.

In order to assess the enzymatic activity of the purified NbSLP1, in-gel zymography was conducted. The results showed that Pro-NbSLP1 and FL-NbSLP1 had no clear colorless bands, indicating no enzymatic activity of NbSLP1 or that the activity was too low for detection by this method (**Figure 2C**). Gel filtration chromatography (HiLoad™ 16/600 Superdex™ 200 pg purchased from GE Healthcare) was adopted to separate mature-NbSLP1-His_6_ from mixture with His_6_-Inhibitor_I9, but the results showed that it failed to remove His_6_-Inhibitor_I9 even in high-resolution fractionation (**Figure S1**), which implied that Inhibitor_I9 may bind to mature NbSLP1 in a non-covalent manner, providing a reasonable clue about low enzymatic activity.

**Figure 3:**
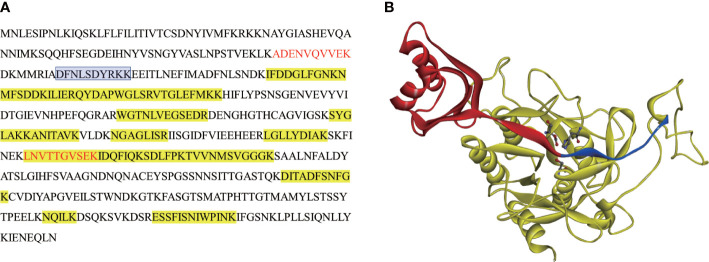
MS analysis and autoproteolysis sites in sequence and predicted 3D structure of NbSLP1. **(A)** Protein sequence of Full-length NbSLP1, peptides of ~14 kDa (band in **Figure 2A** indicated by blue arrow) detected by MS are showed in red font, peptides of ~45 kDa (band in **Figure 2A** indicated by red arrow) highlighted in yellow, N-terminus sequence of mature NbSLP1 is showed in blue frame; **(B)** Predicted 3D structure of NbSLP1. Pro-peptide is showed in red ribbon; mature NbSLP1 is showed in yellow ribbon; autoproteolysis site is showed between red and blue ribbon; NbSLP1 catalytic triad Asp^192^-His^224^-Ser^413^ is showed in ball and stick structure.

N-terminal sequence of mature-NbSLP1 was conducted to identify autoproteolysis sites of NbSLP1 in *E. coli*. The sequencing results showed that N-terminus of mature NbSLP1 is NH_2_-Asp-Phe-Asn-Leu-Ser-Asp-Tyr-Arg-Lys-Lys (DFNLSDYRKK), demonstrating that the cleavage sites is MRIA^104^↓D^105^FNL. To further explore autoproteolysis of NbSLP1 in *E. coli*, pellets at different timepoints post induction by IPTG were collected and sampled to identify autoproteolysis. We found that Pro-NbSLP1 cleaved itself as soon as the protease was detected, 2 hours post induction (**Figure 2D**). According to homologous modeling of NbSLP1 against subtilisin E (3whi.2.A) with SWISS MODEL, cleavage sites were around catalytic triad D^192^H^224^S^413^ in the predicted 3D structure (**Figure 3**). Taken together, these imply autoproteolysis is influenced by catalytic function of the enzyme.

### Mutation in cleavage sites inhibiting autoproteolysis of NbSLP1

**Figure 4:**
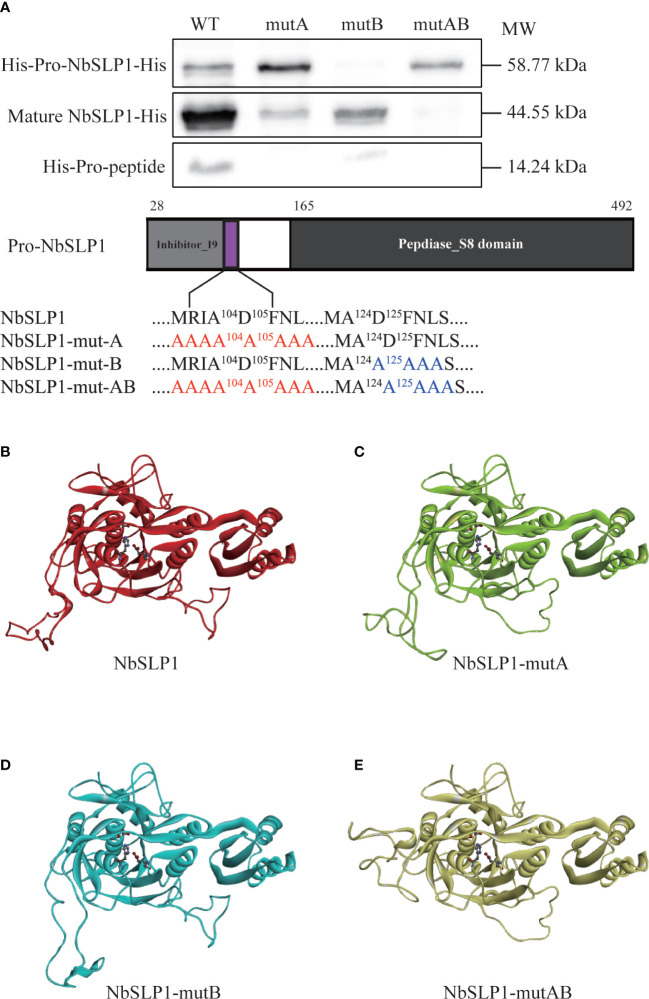
Autoproteolysis analysis of Pro-NbSLP1 mutants in cleavage sites and predicted 3D structure. **(A)** Western blot analysis of Pro-NbSLP1 mutants. Supernatant from induced *E*. *coli* BL21 (DE3) [pET30-*Pro*-*Nbslp1*] (WT), *E*. *coli* BL21 (DE3) [pET30-*Pro*-*Nbslp1-mutA*] (mut A), *E*. *coli* BL21 (DE3) [pET30-*Pro*-*Nbslp1-mutB*] (mut B), *E*. *coli* BL21 (DE3)[pET30-*Pro*-*Nbslp1-mutAB*] (mut AB) were analyzed by immunoblotting using His antibody. Strategy of mutants was showed below. Predicted 3D structure of NbSLP1 and its mutants against Pro-subtilisin E (PDB: 3whi.1) are showed in **B–E**; unmutated NbSLP1 is showed in red ribbon **(B)**, NbSLP1-mut A is showed in green ribbon **(C)**, NbSLP1-mut B is showed in light blue ribbon **(D)**, NbSLP1-mut-AB is showed in yellow ribbon **(E)**.

To further demonstrate autoproteolysis sites of NbSLP1, the expression vector pET-30a containing the site-directed mutagenesis in cleavage sites (M^101^RIADFNL^108^ replaced with A^101^AAAAAAA^108^ named NbSLP1-mut-A) was constructed, and expressed in *E. coli* BL21, while the Western Blotting analysis showed that no effect was observed for the cleavage (**Figure 4**). Due to a similar sequence of mature-SLP1 N-terminus found as M^123^ADFNLSD^130^, combined with the purification results, we suspect that SLP1 may cleave itself more than once. Therefore, another two recombinant plasmids with mutagenesis at sites 125-128 (DFNL) to A^125^AAA^128^, named pET30-*NbSLP1-mut-B*, and both sites 101-108 and 125-128 to A (pET30-*NbSLP1-mut-AB*) were constructed. Western blotting results showed that autoproteolysis of NbSLP1 was inhibited by mutagenesis at two putative cleavage sites, while the homologous model predicted by SWISS MODEL showed no significant shift happened (**Figure 4**).

### Mutation in catalytic triad sites inhibiting autoproteolysis of NbSLP1

**Figure 5:**
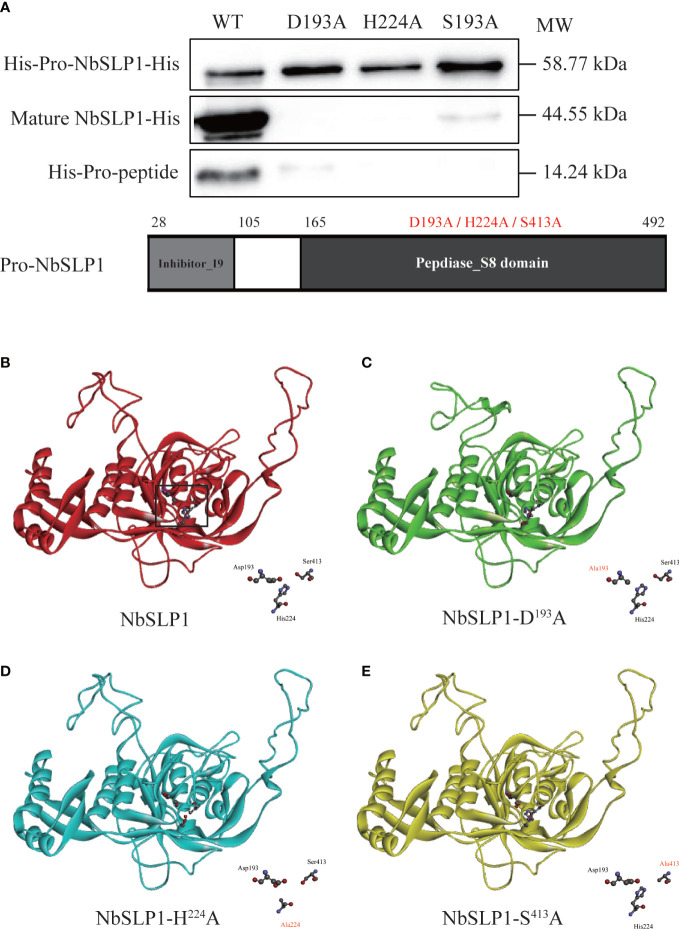
Autoproteolysis analysis of Pro-NbSLP1 mutants of active sites and predicted 3D structure. **(A)** Western blot analysis of Pro-NbSLP1 mutants in active sites. Supernatant from induced *E*. *coli* BL21 (DE3) [pET30-*pro*-*Nbslp1*] (WT), *E*. *coli* BL21 (DE3) [pET30-*pro*-*Nbslp1-D193A*](D193A), *E. coli* BL21 (DE3)[pET30-*pro*-*Nbslp1-H224A*](H224A), *E*. *coli* BL21 (DE3) [pET30-*pro*-*Nbslp1-S413A*](S413A) were analyzed by immunoblotting using His antibody. Details were showed below. Predicted 3D structure of NbSLP1 and its mutants against Pro-subtilisin E are showed in **B–E**; unmutated NbSLP1 is showed in red ribbon **(B)**, NbSLP1-D193A is showed in green ribbon **(C)**, NbSLP1-H224A is showed in blue ribbon **(D)**, NbSLP1-S413A is showed in yellow ribbon **(E)**; catalytic triads were showed in bottom right of each structure.

Mutations in catalytic triad of NbSLP1 were constructed to verify that autoproteolysis of NbSLP1 was induced by catalytic function. NbSLP1 sequence analysis has been performed before, showing that conserved catalytic triad sites of NbSLP1 are D^192^, H^224^ and S^413^, respectively. Homologous models of these mutants predicted by SWISS-MODEL were compared with the model of NbSLP1, showing that there were some changes at catalytic triad. The mutant proteins were expressed at soluble form. Western blotting analysis showed that no cleavage was observed in D193A and H224A mutants, while S413A mutant showed decreased autoproteolysis (**Figure 5**). Among the three catalytic triads, serine nucleophilic attacks the substrate, which is crucial for catalytic activity. The S413A mutant partially maintained the auto-proteolytic ability, demonstrating that catalytic function was the key factor of maturation, but S413A can be cleaved through a mechanism other than autoproteolysis.

## Discussion

### NbSLP1 activated after germination hints at the role in this process

Microsporidia have a unique infective mode as pathogens. However, the molecular mechanisms of spore germination have not been revealed yet. Subtilisin-like protease 1 of *Nosema bombycis* (NbSLP1) can be activated by some conditions after germination, processing itself to release mature-SLP1. Dang *et al.* found that NbSLP1 may have different forms before and after spore germination by dimensional electrophoresis (Dang et al., 2013), implying the role of NbSLP1 in the germination process, while our results shows that NbSLP1 without signal peptide heterologously expressed in *E. coli* can autoprocess itself without any external factor, sharing a similar expression feature with mirolase,the subtilisin-like protease in *Tamberelli forsythia* (Ksiazek et al., 2015), whereas full-length NbSLP1 can be expressed as precursor without any other forms, meaning that signal peptide of NbSLP1 or other factors in *N. bombycis* may work in the enzyme activation during spore germination.

### Activation of NbSLP1 may be related to some stimulus

NbSLP1 localizes in mature spores mostly as a zymogen. We know that activation of zymogen is always related to some environmental factors, such as pH, temperature, and irons. For instance, Furin, the homolog of bacterial subtilisin in mammals, is activated by the pH-dependent mechanism, in which His^69^ in furin propeptide served as pH sensor (Feliciangeli et al., 2006). Inactive furin undergoes auto-cleavage twice, at Arg^107^ and Arg^75^, to cleave and release propeptide, which occurs in the endoplasmic reticulum (ER) and mildly acidic environments (trans-Golgi network), respectively (Anderson et al., 1997; Dillon et al., 2012). Considering the process of expression of this heterogeneous NbSLP1 in *E.coli* cells, there were no such factors that might contribute to activation of NbSLP1. It is reasonable to infer that the cleavage of the enzyme happened spontaneously (auto-cleavage). However, while there was no visible enzymatic activity detected after purification, extra factors might be needed for the full maturation of the protease in spores, such as post-translation regulation (PTM) (Dewpura et al., 2008) and other protein regulations. Meanwhile, there also some stimulus or stability factors related to spore germination, environmental factors (Leitch and Ceballos, 2008), and some proteins like aquaporins (Ghosh et al., 2006) or Septin 2 (Liu et al., 2020), which can also be considered as potential associators of NbSLP1.

### Different forms of mature NbSLP1 may imply various functions

**Figure 6:**
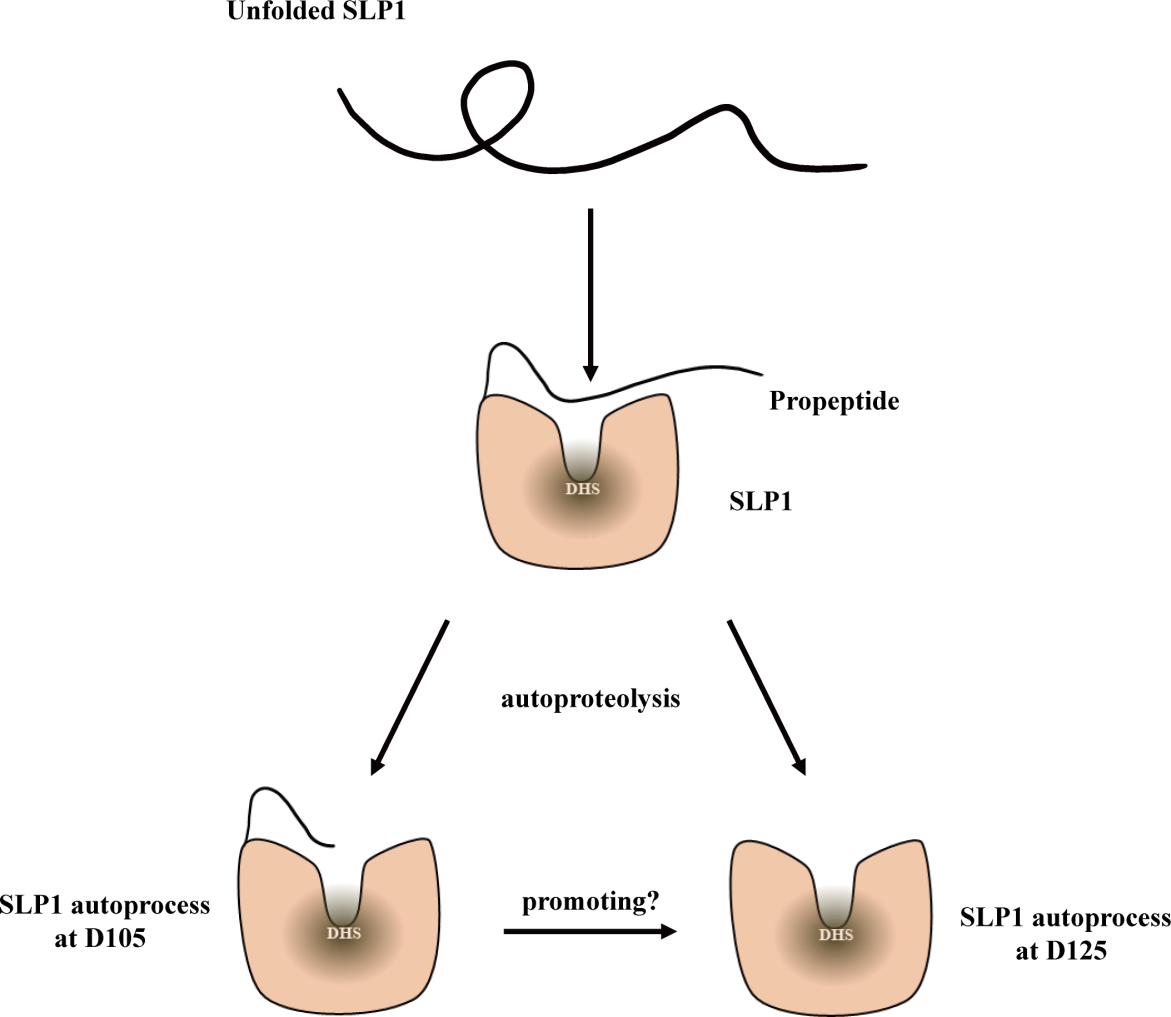
Model of NbSLP1 autoproteolysis. Functional structure of NbSLP1 was constructed correctly with its propeptide; After signal peptide cleavage, at least twice auto-cleavage were observed at Asp^105^ and Asp^125^, respectively, leading to two hypotheses about autoproteolysis of NbSLP1. SLP1 can cleave at Asp^105^ and Asp^125^ at the same time, or SLP1 cleavage itself at Asp^105^ (D105) firstly, and this structure promote the second cleavage at Asp^125^ (D125).

In this study, we focused on the autoproteolysis sites of NbSLP1, and found the cleavage sites at Asp^105^ and Asp^125^, which means at least two forms of mature NbSLP1 expressed *in vitro*, compared with three potential forms of protease after germination *in vivo* (Dang et al., 2013), which demonstrated the variants of NbSLP1. In the process of NbSLP1 autoproteolysis, we speculated that NbSLP1 can cleave at Asp^105^ and Asp^125^ simultaneously, or otherwise the form cleaved at Asp^105^ can promote the second cleavage (**Figure 6**).

Cleavage sites at different locations of the amino acid sequence have been reported in caspase and other subtilisin (Gauthier et al., 2015). Sometimes, different forms of certain protease may lead to other roles. In Caspase-4, there are three cleavage sites at Asp^59^, Asp^270^, and Asp^289^, leading to four forms, named p31, p24, p12 and p10, in which onlythe p33/p10 form is critical for protease activation to induce pyroptosis, while other forms are of little significance in this process (Wang et al., 2020). In view of localization signal and expression pattern of NbSLP1, the protease may work on various substrates in parasites and/or host cells.

### NbSLP1 with propeptide provide foundation for active NbSLP1

Previous studies about NbSLP1 show that it is difficult to produce soluble mature NbSLP1 by the *E. coli* expression system, yeast expression system, and even baculovirus expression vector system. There is some research related to active protease preparation with a subtilisin with its own propeptide (Xie et al., 2019). In this study, we constructed a NbSLP1 vector with its own propeptide to produce the soluble protein. This is because inhibitor_I9 of SLP1 promotes proper folding of mature enzyme, while the pro-peptide also keeps the proenzyme inactive as a temporal inhibitor of SLP (Yabuta et al., 2001). However, the soluble mature NbSLP1 we prepared showed low catalytic activity, which was caused by the rate-limiting process of propeptide released from inactive propeptide-NbSLP1 complex. We are planning to explore the substrate of NbSLP1 to further identify the role of this protease in the parasites spore germination.

In this study, we identified the autoproteolysis and cleavage sites of recombinant Pro-NbSLP1 in position Ala104 and Ala 124, while full-length NbSLP1 dislayed a low cleavage rate. We found that catalytic triad (D^193^H^224^S^413^) and cleavage sites were both significant to NbSLP1 activation, provided that the 3D structure of NbSLP1 and its mutants are less differentiating at some level.

## Data availability statement

The original contributions presented in the study are included in the article. Further inquiries can be directed to the corresponding authors.

## Author contributions

RW and JC designed the project, performed the experiments, analyzed the data, and drafted the manuscript. RW and QL performed most of the experiments. XD, FL, QS, and XS gave input on method refinement. MH performed part of the plasmid construction. BJL review and edit the manuscript. GP and ZZ provided overall study supervision and grant support. All authors read and approved the final manuscript.

## Funding

This study was supported by grants from the Natural Science Foundation of China (Grant No. 31770159; 31602012) and the Academician Fund of Chongqing/Natural Science Foundation of Chongqing (cstc2018jcyj-yszxX0012).

## Acknowledgments

We are grateful to APTBIO who provided MS analysis and N-terminal sequence services and all who providethe free software cited in this article. The authors appreciate Dr. Xianzhi Meng, Qiang He, Qing Lv, Wang Chunxia, and Ni Wenjia for their help on equipment usage, bioinformation analysis, material collection, and inspiration.

## Conflict of interest

The authors declare that the research was conducted in the absence of any commercial or financial relationships that could be construed as a potential conflict of interest.

## Publisher’s note

All claims expressed in this article are solely those of the authors and do not necessarily represent those of their affiliated organizations, or those of the publisher, the editors and the reviewers. Any product that may be evaluated in this article, or claim that may be made by its manufacturer, is not guaranteed or endorsed by the publisher.

## Supplementary material

Supplementary Figure 1 Gel filtration analysis of purified NbSLP1. **(A)** Elution profile of protein samples taken from an absorbance of 280 nm. **(B)** SDS-PAGE analysis of protein samples taken from peak 1, 2 and 3. Mature NbSLP1 are indicated by red arrows and Pro-peptide (Inhibitor_I9 domain) is indicated by blue arrow.

## References

[B1] Alam A. Bhatnagar R. K. Relan U. Mukherjee P. Chauhan V. S. (2013). Proteolytic activity of plasmodium falciparum subtilisin-like protease 3 on parasite profilin, a multifunctional protein. Mol. Biochem. Parasitol. 191 (2), 58–62. doi: 10.1016/j.molbiopara.2013.09.006 24080030

[B2] Anderson E. D. VanSlyke J. K. Thulin C. D. Jean F. Thomas G. (1997). Activation of the furin endoprotease is a multiple-step process: requirements for acidification and internal propeptide cleavage. EMBO J. 16 (7), 1508–1518. doi: 10.1093/emboj/16.7.1508 9130696 PMC1169755

[B3] Barale J. C. Blisnick T. Fujioka H. Alzari P. M. Aikawa M. Braun-Breton C.. (1999). Plasmodium falciparum subtilisin-like protease 2, a merozoite candidate for the merozoite surface protein 1-42 maturase. Proc. Natl. Acad. Sci. U.S.A. 96 (11), 6445–6450. doi: 10.1073/pnas.96.11.6445 10339607 PMC26901

[B4] Dang X. Pan G. Li T. Lin L. Ma Q. Geng L.. (2013). Characterization of a subtilisin-like protease with apical localization from microsporidian nosema bombycis. J. Invertebr. Pathol. 112 (2), 166–174. doi: 10.1016/j.jip.2012.10.009 23178826

[B5] Das S. Hertrich N. Perrin A. J. Withers-Martinez C. Collins C. R. Jones M. L.. (2015). Processing of plasmodium falciparum merozoite surface protein MSP1 activates a spectrin-binding function enabling parasite egress from RBCs. Cell Host Microbe 18 (4), 433–444. doi: 10.1016/j.chom.2015.09.007 26468747 PMC4608996

[B6] Dewpura T. Raymond A. Hamelin J. Seidah N. G. Mbikay M. Chretien M.. (2008). PCSK9 is phosphorylated by a golgi casein kinase-like kinase ex vivo and circulates as a phosphoprotein in humans. FEBS J. 275 (13), 3480–3493. doi: 10.1111/j.1742-4658.2008.06495.x 18498363

[B7] Didier E. S. Weiss L. M. (2011). Microsporidiosis: not just in AIDS patients. Curr. Opin. Infect. Dis. 24 (5), 490–495. doi: 10.1097/QCO.0b013e32834aa152 21844802 PMC3416021

[B8] Dillon S. L. Williamson D. M. Elferich J. Radler D. Joshi R. Thomas G.. (2012). Propeptides are sufficient to regulate organelle-specific pH-dependent activation of furin and proprotein convertase 1/3. J. Mol. Biol. 423 (1), 47–62. doi: 10.1016/j.jmb.2012.06.023 22743102 PMC3444655

[B9] Feliciangeli S. F. Thomas L. Scott G. K. Subbian E. Hung C. H. Molloy S. S.. (2006). Identification of a pH sensor in the furin propeptide that regulates enzyme activation. J. Biol. Chem. 281 (23), 16108–16116. doi: 10.1074/jbc.M600760200 16601116 PMC4293020

[B10] Franzen C. (2004). Microsporidia: how can they invade other cells? Trends Parasitol. 20 (6), 275–279. doi: 10.1016/j.pt.2004.04.009 15147678

[B11] Gauthier M. S. Perusse J. R. Awan Z. Bouchard A. Tessier S. Champagne J.. (2015). A semi-automated mass spectrometric immunoassay coupled to selected reaction monitoring (MSIA-SRM) reveals novel relationships between circulating PCSK9 and metabolic phenotypes in patient cohorts. Methods 81, 66–73. doi: 10.1016/j.ymeth.2015.03.003 25770357

[B12] Ghosh K. Cappiello C. D. McBride S. M. Occi J. L. Cali A. Takvorian P. M.. (2006). Functional characterization of a putative aquaporin from encephalitozoon cuniculi, a microsporidia pathogenic to humans. Int. J. Parasitol. 36 (1), 57–62. doi: 10.1016/j.ijpara.2005.08.013 16197948 PMC3086640

[B13] Han B. Weiss L. M. (2017). Microsporidia: Obligate intracellular pathogens within the fungal kingdom. Microbiol. Spectr. 5 (2):10.1128/microbiolspec.FUNK-0018-2016. doi: 10.1128/microbiolspec.FUNK-0018-2016 PMC5613672 28944750

[B14] Howell M. Dumitrescu D. G. Blankenship L. R. Herkert D. Hatzios S. K. (2019). Functional characterization of a subtilisin-like serine protease from vibrio cholerae. J. Biol. Chem. 294 (25), 9888–9900. doi: 10.1074/jbc.RA119.007745 31076508 PMC6597830

[B15] Keeling P. J. Fast N. M. (2002). Microsporidia: biology and evolution of highly reduced intracellular parasites. Annu. Rev. Microbiol. 56, 93–116. doi: 10.1146/annurev.micro.56.012302.160854 12142484

[B16] Ksiazek M. Karim A. Y. Bryzek D. Enghild J. J. Thogersen I. B. Koziel J.. (2015). Mirolase, a novel subtilisin-like serine protease from the periodontopathogen tannerella forsythia. Biol. Chem. 396 (3), 261–275. doi: 10.1515/hsz-2014-0256 25391881 PMC4682893

[B17] Lantz M. S. Ciborowski P. (1994). Zymographic techniques for detection and characterization of microbial proteases. Methods Enzymol. 235, 563–594. doi: 10.1016/0076-6879(94)35171-6 8057927

[B18] Leitch G. J. Ceballos C. (2008). Effects of host temperature and gastric and duodenal environments on microsporidia spore germination and infectivity of intestinal epithelial cells. Parasitol. Res. 104 (1), 35–42. doi: 10.1007/s00436-008-1156-4 18751726 PMC2737319

[B19] Liu F. Chen J. Dang X. Meng X. Wang R. Bao J.. (2020). Nbseptin2 expression pattern and its interaction with NbPTP1 during microsporidia nosema bombycis polar tube extrusion. J. Eukaryot. Microbiol. 67 (1), 45–53. doi: 10.1111/jeu.12752 31332864

[B20] Liu F. Ma Q. Dang X. Wang Y. Song Y. Meng X.. (2017). Identification of a new subtilisin-like protease NbSLP2 interacting with cytoskeletal protein septin in microsporidia nosema bombycis. J. Invertebr. Pathol. 148, 110–117. doi: 10.1016/j.jip.2017.06.004 28625841

[B21] Mai H. N. Cruz-Flores R. Aranguren Caro L. F. White B. N. Dhar A. K. (2020). A comparative study of enterocytozoon hepatopenaei (EHP) challenge methods in penaeus vannamei. J. Invertebr. Pathol. 171, 107336. doi: 10.1016/j.jip.2020.107336 32044360

[B22] Oh Y. Donofrio N. Pan H. Coughlan S. Brown D. E. Meng S.. (2008). Transcriptome analysis reveals new insight into appressorium formation and function in the rice blast fungus magnaporthe oryzae. Genome Biol. 9 (5), R85. doi: 10.1186/gb-2008-9-5-r85 18492280 PMC2441471

[B23] Ronnebaumer K. Wagener J. Gross U. Bohne W. (2006). Identification of novel developmentally regulated genes in encephalitozoon cuniculi: an endochitinase, a chitin-synthase, and two subtilisin-like proteases are induced during meront-to-sporont differentiation. J. Eukaryot. Microbiol. 53 Suppl 1, S74–S76. doi: 10.1111/j.1550-7408.2006.00179.x 17169074

[B24] Saitoh H. Fujisawa S. Ito A. Mitsuoka C. Berberich T. Tosa Y.. (2009). SPM1 encoding a vacuole-localized protease is required for infection-related autophagy of the rice blast fungus magnaporthe oryzae. FEMS Microbiol. Lett. 300 (1), 115–121. doi: 10.1111/j.1574-6968.2009.01769.x 19765082

[B25] Saouros S. Dou Z. Henry M. Marchant J. Carruthers V. B. Matthews S. (2012). Microneme protein 5 regulates the activity of toxoplasma subtilisin 1 by mimicking a subtilisin prodomain. J. Biol. Chem. 287 (43), 36029–36040. doi: 10.1074/jbc.M112.389825 22896704 PMC3476271

[B26] Seawell N. A. Bose J. L. (2021). Analysis of murein hydrolases and proteases through zymography. Methods Mol. Biol. 2341, 9–16. doi: 10.1007/978-1-0716-1550-8_2 34264455

[B27] Takagi H. Koga M. Katsurada S. Yabuta Y. Shinde U. Inouye M.. (2001). Functional analysis of the propeptides of subtilisin e and aqualysin I as intramolecular chaperones. FEBS Lett. 508 (2), 210–214. doi: 10.1016/s0014-5793(01)03053-8 11718717

[B28] Tang W. Bobeica S. C. Wang L. van der Donk W. A. (2019). CylA is a sequence-specific protease involved in toxin biosynthesis. J. Ind. Microbiol. Biotechnol. 46 (3-4), 537–549. doi: 10.1007/s10295-018-2110-9 30484123 PMC6450559

[B29] Timofeev S. A. Sendersky I. V. Pavlova O. A. Dolgikh V. V. (2014). Peculiarities of the expression, structure, and localization of the subtilisin-like protease in the microsporidium paranosema locustae. Parazitologiia 48 (5), 337–347. 25929105

[B30] Wang K. Sun Q. Zhong X. Zeng M. Zeng H. Shi X.. (2020). Structural mechanism for GSDMD targeting by autoprocessed caspases in pyroptosis. Cell 180 (5), 941–955.e920. doi: 10.1016/j.cell.2020.02.002 32109412

[B31] Wanyiri J. W. Techasintana P. O'Connor R. M. Blackman M. J. Kim K. Ward H. D. (2009). Role of CpSUB1, a subtilisin-like protease, in cryptosporidium parvum infection *in vitro* . Eukaryot. Cell 8 (4), 470–477. doi: 10.1128/EC.00306-08 19168760 PMC2669210

[B32] Withers-Martinez C. Jean L. Blackman M. J. (2004). Subtilisin-like proteases of the malaria parasite. Mol. Microbiol. 53 (1), 55–63. doi: 10.1111/j.1365-2958.2004.04144.x 15225303

[B33] Xie G. Shao Z. Zong L. Li X. Cong D. Huo R. (2019). Heterologous expression and characterization of a novel subtilisin-like protease from a thermophilic thermus thermophilus HB8. Int. J. Biol. Macromol. 138, 528–535. doi: 10.1016/j.ijbiomac.2019.07.101 31323269

[B34] Yabuta Y. Takagi H. Inouye M. Shinde U. (2001). Folding pathway mediated by an intramolecular chaperone: propeptide release modulates activation precision of pro-subtilisin. J. Biol. Chem. 276 (48), 44427–44434. doi: 10.1074/jbc.M107573200 11577106

