# Supplementary figures and images for: Maturation of subtilisin-like protease NbSLP1 from microsporidia Nosema bombycis
Source: Front Cell Infect Microbiol. 2022 Aug 15;12:897509. doi: 10.3389/fcimb.2022.897509 (PMC9421246; doi:10.3389/fcimb.2022.897509)

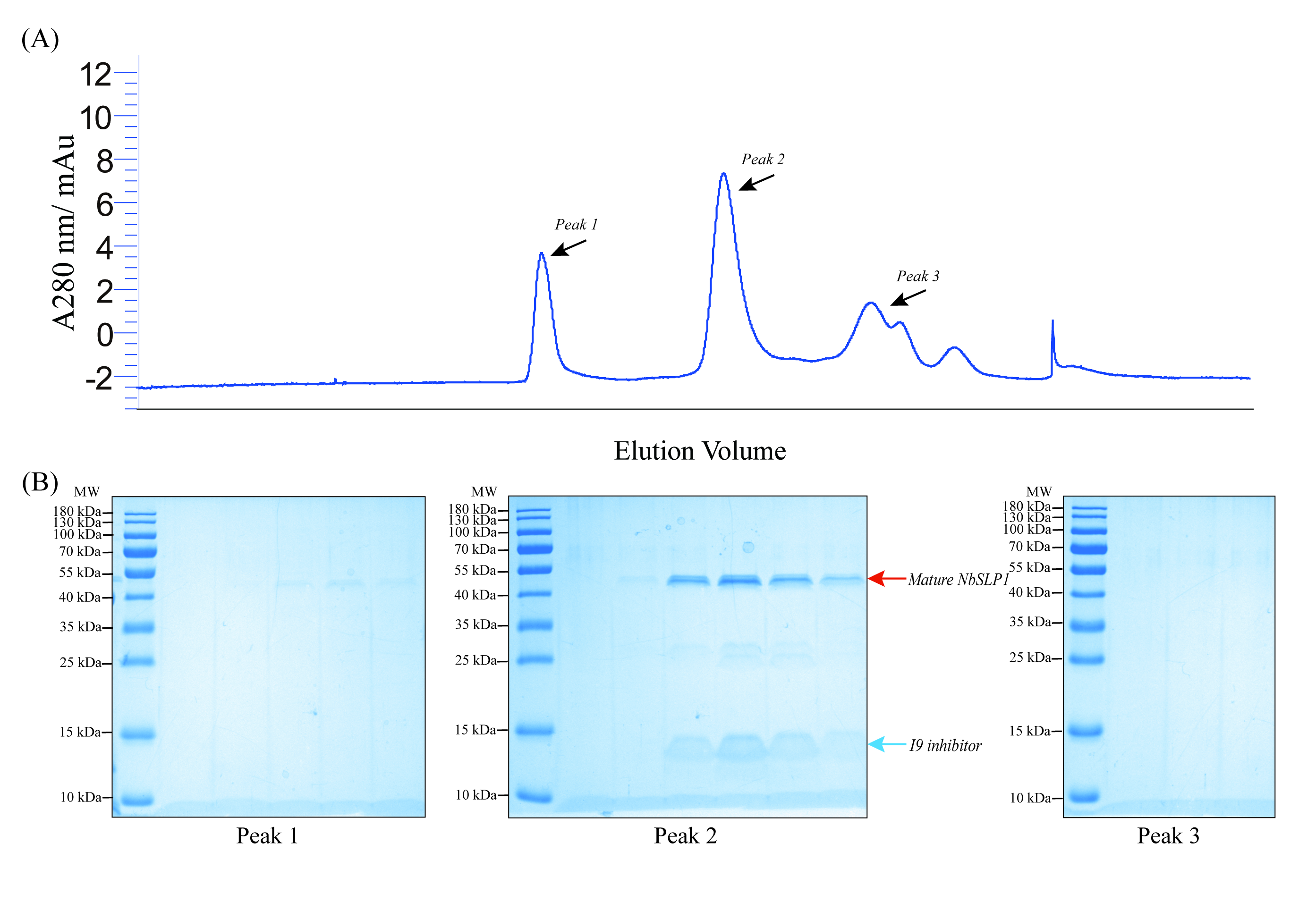

Supplement: Supplementary Figure 1 — Gel filtration analysis of purified NbSLP1. (A) Elution profile of protein samples taken from an absorbance of 280 nm. (B) SDS-PAGE analysis of protein samples taken from peak 1, 2 and 3. Mature NbSLP1 are indicated by red arrows and Pro-peptide (Inhibitor_I9 domain) is indicated by blue arrow. [file Image_1.tif]
